# Ultra-high throughput functional enrichment of large monoamine oxidase (MAO-N) libraries by fluorescence activated cell sorting†
†Electronic supplementary information (ESI) available. See DOI: 10.1039/c8an00851e


**DOI:** 10.1039/c8an00851e

**Published:** 2018-09-10

**Authors:** Joanna C. Sadler, Andrew Currin, Douglas B. Kell

**Affiliations:** a School of Chemistry , The University of Manchester , 131 Princess St , Manchester M1 7DN , UK; b The Manchester Institute of Biotechnology , The University of Manchester , 131 Princess St , Manchester M1 7DN , UK; c Centre for the Synthetic Biology of Fine and Speciality Chemicals (SYNBIOCHEM) , The University of Manchester , 131 Princess St , Manchester M1 7DN , UK . Email: joanna.sadler@manchester.ac.uk ; Email: andrew.currin@manchester.ac.uk ; Email: dbk@manchester.ac.uk ; http://dbkgroup.org/@dbkell

## Abstract

Directed evolution enables the improvement and optimisation of enzymes for particular applications and is a valuable tool for biotechnology and synthetic biology. However, studies are often limited in their scope by the inability to screen very large numbers of variants to identify improved enzymes. One class of enzyme for which a universal, operationally simple ultra-high throughput (>10^6^ variants per day) assay is not available is flavin adenine dinucleotide (FAD) dependent oxidases. The current high throughput assay involves a visual, colourimetric, colony-based screen, however this is not suitable for very large libraries and does not enable quantification of the relative fitness of variants. To address this, we describe an optimised method for the sensitive detection of oxidase activity within single *Escherichia coli* (*E. coli*) cells, using the monoamine oxidase from *Aspergillus niger*, MAO-N, as a model system. In contrast to other methods for the screening of oxidase activity *in vivo*, this method does not require cell surface expression, emulsion formation or the addition of an extracellular peroxidase. Furthermore, we show that fluorescence activated cell sorting (FACS) of large libraries derived from MAO-N under the assay conditions can enrich the library in functional variants at much higher rates than *via* the colony-based method. We demonstrate its use for directed evolution by identifying a new mutant of MAO-N with improved activity towards a novel secondary amine substrate. This work demonstrates, for the first time, an ultra-high throughput screening methodology widely applicable for the directed evolution of FAD dependent oxidases in *E. coli*.

## Abstract 2

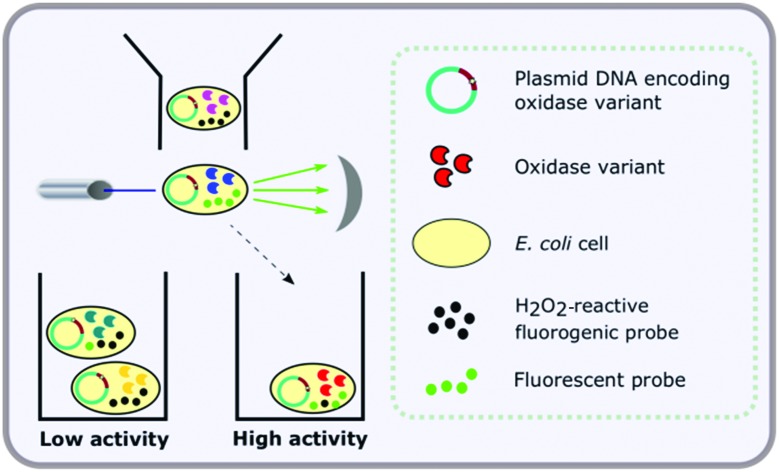
A novel ultra-high throughput screen for *in vivo* detection of oxidase activity in *E. coli* cells and its application to directed evolution.

## Introduction

Directed evolution (DE) is a powerful tool for the improvement and optimisation of enzymes and has been widely adopted throughout the field of biotechnology.^1^–^5^ For example, engineered enzymes have been employed in the synthesis of pharmaceuticals,^4^,^6^,^7^ biofuels,^8^,^9^ detergents,^10^ animal feed supplements^11^,^12^ and biosensors.^13^ A typical DE workflow comprises three main stages: (1) identification of a suitable parent enzyme; (2) creation of genetic diversity; and (3) selection or screening for the variants with improved fitness. This cycle may be performed iteratively until a protein with the required fitness is obtained. While it is accepted that large, diverse variant libraries are required to navigate the vast fitness landscape intelligently,^14^,^15^ a lack of robust methods to screen or select from large numbers (>10^6^) of variants efficiently represents a major bottleneck in many DE projects. To address this, ultra-high throughput screening techniques have been developed using fluorescence activated cells sorting (FACS),^16^–^21^ fluorescence activated droplet sorting (FADS)^22^,^23^ and microchamber arrays coupled with optical or robotic extraction.^24^,^25^

Despite these advances, there are still many classes of biotechnologically important enzymes for which a generally applicable, ultra-high throughput assay is not available. One such example is that of flavin adenine dinucleotide (FAD) dependent oxidases, representing a large and diverse class of enzymes which catalyse the removal of a hydride equivalent from the substrate and release the oxidised product, producing hydrogen peroxide as a by-product. These enzymes use molecular oxygen to regenerate the cofactor *in situ*, making them operationally simple and economically viable for use in industrial settings and, as such, a popular target for DE. In particular, the monoamine oxidase from *Aspergillus niger* (MAO-N)^26^–^30^ has been heavily engineered to oxidise a wide range of primary, secondary and even tertiary amines.^31^–^36^ When coupled with a chemical reducing agent, a dynamic kinetic resolution can be achieved to yield enantiomerically pure amines, which are valuable building blocks for many fine and speciality chemicals, including many pharmaceuticals.^37^,^38^ The most widely used screening assay for MAO-N and variants thereof involves a colony-based screen in which colonies form a red colour when amine-oxidising enzyme activity produces H_2_O_2_ in the presence of an enzyme substrate, a chromogenic peroxidase substrate and an added (extracellular) peroxidase.^37^,^39^ However, for the analysis of large (>10^5^) variant libraries that explore a wider area of sequence space, a higher throughput assay is required. Furthermore, as *in silico* approaches to protein engineering become increasingly popular, there is a need to develop methods for the quantification of the relative fitness of variants in a library, which is not catered for by the existing colony-based screen.^14^,^40^–^42^

While there have been no reports of an ultra-high throughput screening and sorting assay for MAO-N variants to date, two FACS assays for the directed evolution of FAD dependent glucose oxidase (GOx) in yeast have been reported.^43^,^44^ Both of these assays were based on a yeast surface expression system in a single or double emulsion. The first employs a vanadium bromoperoxidase-coupled fluorescence reporter in a double emulsion to detect hydrogen peroxide production,^44^ while the second uses horseradish peroxidase (HRP) and a fluorescein precursor in a single emulsion to couple GOx activity to a fluorescent output.^43^ In both cases, FACS of the droplets resulted in enrichment of the variant libraries in active clones. Despite these advances, the requirement for yeast surface expression, addition of an exogenous peroxidase and the delivery of substrates into the droplets limits the widespread application of these assays for the DE of other oxidases.

**Fig. 1:**
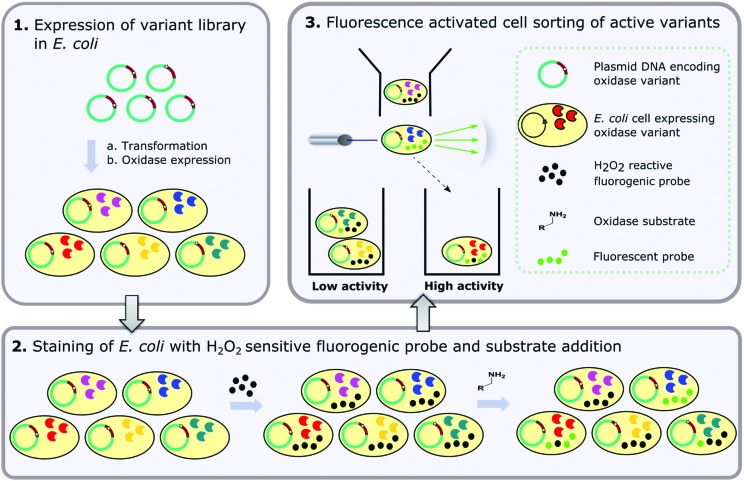
Overview of the flow cytometric assay for detection of amine oxidase activity in *E. coli* with the three main steps comprising (1) expression of the amine oxidase variant library in *E. coli*; (2) staining of *E. coli* cells with a fluorogenic probe which is sensitive to oxidation by H_2_O_2_ in the presence of an intracellular, endogenous peroxidase and (3) fluorescence activated cell sorting (FACS) of active variants based on fluorescence of the oxidised probe.

Given that a number of fluorogenic dyes for intracellular detection of reactive oxygen species (ROS) are commercially available, we were motivated to develop a method that could be used without the need for yeast cell-surface expression and droplet formation, using *E. coli* as a host. The present paper describes the development and optimisation of just such an assay and its application to identify improved variants through directed evolution. The overall process is shown in Fig. 1. First, *E. coli* cells are transformed with DNA encoding the enzyme of interest, or a variant library thereof, and enzyme expression is induced, such that each cell harbours a single variant. Next, the induced cells are treated with a fluorogenic probe which is sensitive to oxidation by H_2_O_2_ in the presence of an endogenous, intracellular peroxidase. When the amine substrate is added, cells expressing active variants exhibit higher fluorescence due to the production of more H_2_O_2_ and are sorted by FACS. We illustrate the utility of this process by employing the method to identify a mutant of MAO-N D5^26^,^37^ exhibiting activity towards a substrate for which no activity is observed for the parent enzyme. Although demonstrated for MAO-N, this method could be applied to the analysis of any enzyme that produces H_2_O_2_ as a by-product of its reaction.

## Materials and methods

### Preparation of MAO-N D5 gene construct and variant libraries

The codon optimised gene for MAO-N D5 was prepared using GeneGenie and SpeedyGenes as previously described^5^–^7^ and cloned into the pBbE2K expression vector,^39^ such that the encoded protein contains an N-terminal His_10_-tag. Variants were created using asymmetric PCR mutagenesis as previously described^45^,^46^ and cloned into the pBbE2K vector, with the resulting library encoding 40 000 variants. All cloning reactions were performed using the In-Fusion cloning kit (Takara Clontech) according to the supplier's protocol.

### Preparation of cell samples (single clones)

Chemically competent cells (T7 Express and 5-alpha) were purchased from New England Biolabs. 25 μL *E. coli* cells were transformed with 1–5 ng plasmid DNA according to the supplier's protocol. 3 mL of Terrific Broth (TB) containing 0.4% (v/v) glycerol and 50 μg mL^–1^ kanamycin was inoculated with a single colony from the transformation and incubated at 37 °C, 180 rpm (5.1 cm orbit diameter) and grown to an OD_600_ of 0.6 before inducing protein expression by the addition of anhydrotetracycline (as a 20 μM solution in ethanol) to a final concentration of 200 nM. Cultures were then incubated at 30 °C, 180 rpm (5.1 cm orbit diameter) for 4 hours before harvesting the cells by centrifugation (9500*g*, 5 minutes at 4 °C). The supernatant was discarded and the cell pellet stored at –80 °C for at least 3 hours prior to analysis by flow cytometry. This freeze–thaw step is crucial for reproducible probe uptake and cell fluorescence.

### Preparation of cell samples (variant libraries)

25 μL *E. coli* cells (T7 Express) were transformed with 1–5 ng plasmid DNA or 1 μL cloning reaction according to the supplier's protocol. 3 mL of Lysogeny Broth (LB) containing 50 μg mL^–1^ kanamycin was inoculated with 400 μL outgrowth (SOC medium) culture and incubated at 37 °C overnight. 3 mL TB containing 0.4% (v/v) glycerol and 50 μg mL^–1^ kanamycin was inoculated with 1% (v/v) overnight culture and incubated at 37 °C, 180 rpm (5.1 cm orbit diameter) to an OD_600_ of 0.6. Protein expression was induced and cells collected as described above for single clones.

### Analysis by flow cytometry

Cell pellets were thawed on ice and resuspended in 100 mM, pH 6.2 potassium phosphate buffer containing 100 mM KCl, 3 mM MgCl_2_ and 1% (v/v) butan-1-ol, to an OD_600_ of 1. C-H_2_DCFDA (as a 10 mM solution in DMSO) was added to a final concentration of 250 μM and the cells were incubated at 37 °C, 180 rpm (5.1 cm orbit diameter) for 1 hour. The cells were harvested by centrifugation (9500*g*, 5 minutes, 4 °C) and the supernatant discarded. The cell pellet was resuspended in 50 mM potassium phosphate buffer containing 100 mM KCl and 3 mM MgCl_2_, pH 7.5 and the reaction initiated by the addition of the amine substrate (as a 100 mM solution in 100 mM potassium phosphate buffer containing 100 mM KCl and 3 mM MgCl_2_) to a final concentration of 10 mM. The substrates used were (*S*)-(–)-α-methylbenzylamine (AMBA), amylamine (AA), benzylamine (BZA), butylamine (BTA), cyclohexylamine (CHA), or *N*-(4-methoxybenzyl)ethenamine (MBEA). Dyes and substrates were purchased from Sigma-Aldrich, Thermo Fisher Scientific or Enamine and used without further purification. Catalase from bovine liver (to a final concentration of 0.01 mg mL^–1^) was added at the time of substrate addition to scavenge H_2_O_2_ in the extracellular media, thereby minimising potential ‘cross-talk’ between cells. The reaction was quenched by the addition of ascorbic acid (as a 100 mM solution in Milli-Q water) to a final concentration of 10 mM. The flow cytometric cell sorter used was the Sony SH800S and the fluorescein product was excited by a single laser operating at 488 nm and detecting fluorescence in the FL2 channel (525/50 nm). Forward scatter (FSC) and side scatter (SSC) data were collected with detection thresholds of 2% and 30%, respectively and data output in files obeying the FCS 3.0 standard.^41^ Cells were gated on FSC-A *vs.* SSC-A and singlets identified by sub-gating on FSC-A *vs.* FSC-H. Flow cytometric data were analysed and figures were prepared using the FlowJo v10 software package.

### Functional enrichment of libraries by FACS

For isolation of functional variants from MAO-N D5 libraries, 1000 cells in the 99^th^ percentile of the FL2-H channel were sorted into an empty collection tube. Amplification of the gene variants was performed by PCR containing 25 μL KAPA HiFi HotStart ReadyMix (Roche Diagnostics), 4 μL sorted cells from FACS, 500 nM forward primer (from 10 μM stock), 500 nM reverse primer (from 10 μM stock) and Milli-Q water to a final volume of 50 μL. The PCR was thermocycled as follows: 95 °C for 5 minutes, then 35 cycles of 98 °C for 20 seconds, 65 °C for 15 seconds and 72 °C for 40 seconds, followed by 72 °C for 2 minutes. The amplified MAO-N genes were purified by gel extraction using a NucleoSpin DNA purification kit (Macherey-Nagel) according to the manufacturer's protocol, eluting in 20 μL elution buffer. The resulting genes were cloned into the pBbE2K expression vector^39^ using the In-Fusion cloning kit (Takara Clontech) and gene expression performed as described above for variant libraries.

## Results

### Assay development

#### Probe selection

**Fig. 2:**
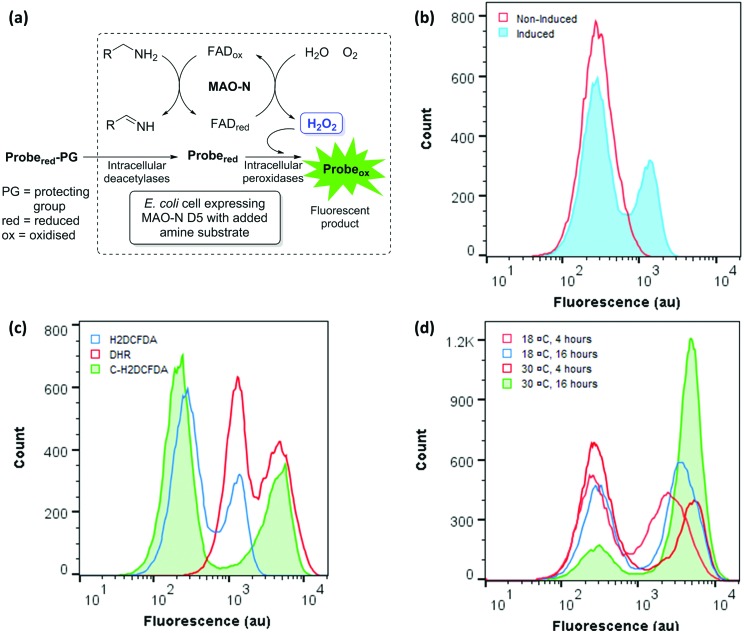
(a) Coupling of MAO-N activity to fluorescence *in vivo*. (b) FCM analysis of induced and non-induced *E. coli* cells containing the MAO-N expression construct, treated with H_2_DCFDA and incubated with AMBA. (c) Comparison of fluorogenic dyes: FCM analysis of *E. coli* cells expressing MAO-N D5 stained with H_2_DHDCF, C-H_2_DHDCF or DHR and incubated with AMBA. (d) Optimisation of conditions for the expression of MAO-N D5 in *E. coli*: FCM analysis of *E. coli* cells incubated at 18 °C or 30 °C for 4 or 16 hours after inducing MAO-N D5 expression.

A number of non-fluorescent ‘dihydro’ dyes can be oxidised in the presence of various ROS to form fluorescent derivatives (Fig. 2a).^47^–^49^ These include 2′,7′-dichlorodihydrofluorescein diacetate (H_2_DCFDA), carboxy-2′,7′-dichlorodihydrofluorescein diacetate (C-H_2_DCFDA) (which both require deacetylation by endogenous esterases prior to oxidation) and dihydrorhodamine123 (DHR). Initially, the feasibility of using these dyes to detect oxidase activity *in vivo* was investigated by incubating MAO-N-induced and non-induced cells with H_2_DCFDA and the MAO-N substrate (*S*)-α-methylbenzylamine (AMBA) (Fig. 2b). When both substrate and dye were present in high concentration (10 mM and 50 μM, respectively), a second population with higher fluorescence than that of the non-induced negative control was detected. The absence of this second population in the non-induced control indicates the observed increase in fluorescence for the induced sample is specific to MAO-N activity. Specificity of the fluorescence was further confirmed by analysis of induced cells incubated with C-H_2_DCFDA in the absence of amine substrate. In this case, a small shift in fluorescence of the whole population was observed compared to induced cells only, and second population (fluorescence ∼1 log unit higher) was only observed when the amine substrate was added (Fig. S1a†).

Encouraged by these data, three ‘dihydro’ dyes H_2_DCFDA, C-H_2_DCFDA and DHR were tested with induced and non-induced cells (Fig. 2c). While DHR was ineffective at distinguishing between the induced and non-induced samples, both H_2_DCFDA and C-H_2_DCFDA successfully reported a distinct second population for the induced sample, which is hereafter referred to as the ‘positive’ population. Cells displaying lower fluorescence (10^2^–10^3^ au) are hereafter referred to as the ‘negative population’. C-H_2_DCFDA was selected for further development as it displayed a higher fluorescence for the positive population relative to the negative population, which would enhance resolution during sorting experiments. The effect of induction time and temperature on the ratio of the two populations was also investigated, with the aim of maximising the proportion of cells in the positive population relative to the negative population (Fig. 2d). A higher proportion of the cells gave a positive fluorescence reading following a 4-hour induction than those that had been incubated overnight, with a 30 °C post induction temperature resulting in 85% of the total gated population exhibiting a ‘positive’ readout relative to the non-induced control.

### Optimisation of assay conditions

**Fig. 3:**
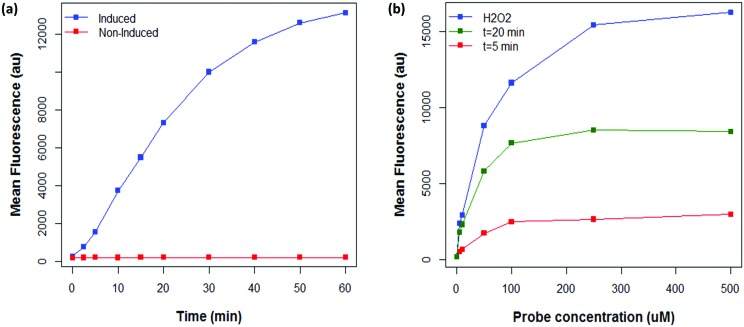
(a) Effect of substrate incubation time on fluorescence of *E. coli* cells expressing MAO-N D5 stained with 50 μM C-H_2_DCFDA and incubated with AMBA, compared to a non-induced control. (b) Effect of probe concentration on the maximum fluorescence of *E. coli* cells stained with C-H_2_DCFDA determined by the addition of 10 mM H_2_O_2_ (blue line) prior to analysis, in comparison to the fluorescence of C-H_2_DCFDA stained cells after incubation with AMBA for 5 (red line) and 20 (green line) minutes.

Having obtained proof of concept and identified a suitable ROS probe, we sought to determine the linear range of the assay. A time course experiment was performed, in which the fluorescence of *E. coli* cells expressing MAO-N was measured for 60 minutes after substrate (AMBA) addition (Fig. 3a). A linear increase in mean fluorescence was observed over the first 30 minutes, followed by a slower rate of increase up to 1 hour after substrate addition. No further increase in fluorescence was observed after 1 hour (data not shown). By contrast, no change in fluorescence of non-induced cells was observed over 60 minutes, further confirming the specificity of the fluorescence in the induced samples.

It was hypothesised that the linear range of the assay may be increased by using a higher concentration of C-H_2_DCFDA, enabling faster mutants to be detected before the system became saturated. To this end, the maximum fluorescence of *E. coli* cells expressing MAO-N stained with a range of concentrations of C-H_2_DCFDA was measured by spiking samples with exogenous H_2_O_2_, which rapidly saturates the available probe such that cells exhibit the maximum possible fluorescence. Additionally, cells stained with each concentration of C-H_2_DCFDA were analysed after 5 and 20 minutes incubation time with the substrate AMBA (Fig. 3b). These data revealed 250 μM C-H_2_DCFDA to provide ∼2 fold higher maximum fluorescence than the initial conditions (50 μM C-H_2_DCFDA). At this higher probe concentration, cells were within the linear range of the assay after 20 minutes incubation with AMBA.

Given the time sensitivity of the assay, we were motivated to screen ROS scavenging agents to stop the assay prior to analysis, thereby improving operational simplicity (*i.e.* time taken to analyse samples) and increasing accuracy. Based on related publications, both ascorbic acid and sodium pyruvate were investigated.^50^,^51^ Out of these, ascorbic acid at a final concentration of 10 mM was found to quench the assay effectively at the desired time-point, such that the cells could be analysed up to an hour after quenching with no change in fluorescence.

Analysis of the same expression construct (MAO-N pBbE2K) in different host strains under the optimised assay conditions identified *E. coli* K12 cells to have high heterogeneity and exhibit an increase in fluorescence for the non-induced sample. In contrast, B strain *E. coli* cells showed less heterogeneity and lower background fluorescence and therefore were used in all further experiments.

We found that a freeze–thaw step was crucial for successful reproduction of the assay. When the freeze–thaw step was omitted, no positive population was observed for induced cells under the assay conditions. Furthermore, no shift in fluorescence was observed for induced or non-induced cells after spiking with exogenous H_2_O_2_. Based on these data, we consider that the C-H_2_DCFDA enters the cells *via* holes in the cell membrane caused by the freeze–thaw cycle (rather than *via* any transporter proteins in the cell membrane^52^,^53^). Cell permeability was improved by addition of butan-1-ol in the staining buffer used to resuspend cells after the freeze–thaw,^54^ with a final concentration of 1% (v/v) being found to give optimum results (data not shown).

**Fig. 4:**
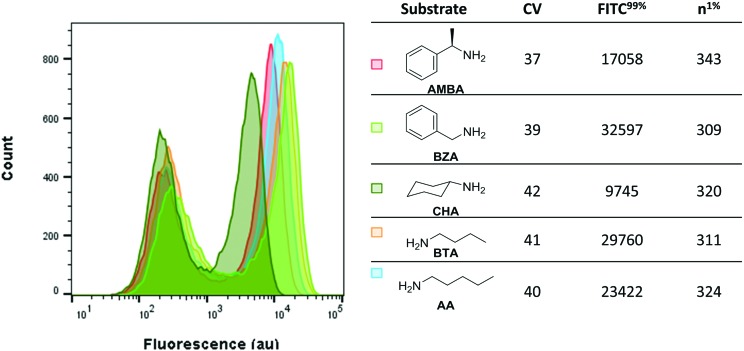
Fluorescence of C-H_2_DCFDA stained *E. coli* incubated with a library of amine substrates. Data were collected after 10 minutes incubation time in each case and was gated on FSC-A *vs.* SSC-A. *n*^1%^: Number of cells with fluorescence in the 99% percentile.

Next, we investigated whether the assay could detect varying levels of activity of MAO-N D5 with a panel of primary amine substrates. To this end, the fluorescence of *E. coli* expressing MAO-N D5 under the assay conditions and incubated with each of the five amine substrates was analysed (Fig. 4). Consistent with the findings on substrate specificity seen in previous reports,^33^,^36^ induced cells incubated with CHA had the lowest fluorescence, followed by AMBA and then AA. As predicted from the *in vitro k*_cat_ values, induced cells incubated with BTA and BZA had the highest fluorescence. These data validate this method as a simple and sensitive method for screening enzyme activity against a panel of substrates, which will have uses in mapping structure–activity relationships in future studies.

### Screening and identification of improved enzyme variants

**Fig. 5:**
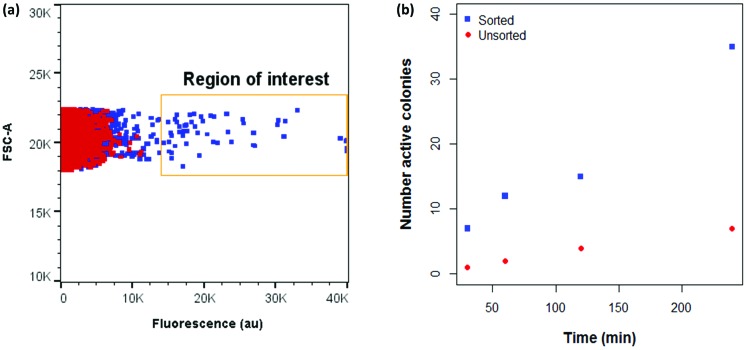
(a) Comparison between fluorescence of CASTing library and MAO-N D5 with MBEA under FCM assay conditions. (b) Functional enrichment of a library of MAO-N variants incubated with MBEA after two rounds of sorting. ROI: Region of interest.

A major advantage of the flow cytometric assay to analyse oxidase activity in comparison to the solid phase assay is the ability to quickly analyse large numbers of variants (our instrument measures up to 10 000 cells per second). This is particularly useful in directed evolution projects, where it is necessary to screen large variant libraries in order to navigate vast and rugged sequence spaces efficiently.^14^ Coupling flow cytometry to FACS enables the isolation of cells with high fluorescence and, as MAO-N variants with a higher turnover number are expected to display higher fluorescence under the assay conditions, FACS can be utilised to isolate improved variants. To test this hypothesis, a combinatorial active site testing (CASTing) library of 40 000 variants was prepared as previously described,^39^,^55^–^58^ and screened for activity using the flow cytometric (FCM) assay using the secondary amine *N*-(4-methoxybenzyl)ethenamine (MBEA) as a substrate, which is not oxidised by MAO-N D5, D9 or D11.^50^ Using the D5 variant as the negative baseline, cells from the library with elevated fluorescence were sorted and subjected to a second round of screening (Fig. 5a).

In initial experiments, sorted cells were out-grown in LB medium and the resultant culture used as inoculant for an expression culture to prepare samples of the sorted variants. However, in all cases, a single, negative population was observed on the second round of analysis. This suggests that cells harbouring active MAO-N variants cannot grow at the same rate as negative cells following FACS, thus leading to a dominance of negative variants in the culture. Further experiments revealed that for efficient recovery of improved variants it was necessary to amplify the sorted MAO-N variants’ DNA by polymerase chain reaction (PCR), ligate into the expression vector and transform into fresh *E. coli* cells for expression and re-analysis using the FCM assay.

**Fig. 6:**
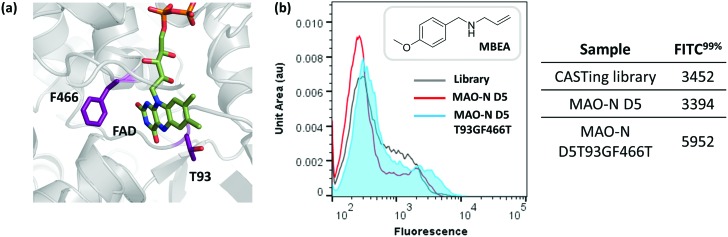
(a) Flow cytometric analysis of MAO-N D5 T93GF466T activity with MBEA in comparison to MAO-N D5 and the variant library under assay conditions. (b) Position of T93 and F466 relative to the cofactor, FAD, in the active site of MAO-N D5 (PBD accession code: 2VVM).

After two iterative rounds of sorting, sorted variants were analysed by the previously described solid phase screen^37^,^39^ and the number of active colonies compared to that for the unsorted CASTing library was counted. As shown in Fig. 5b, ∼5–7× more colonies from the sorted displayed oxidase activity towards MBEA than from the unsorted library, indicating functional enrichment through the FACS assay. The most active clone from the solid phase screen of the sorted CASTing library was identified as the double mutant MAO-N D5 T93G/F466T, with both mutations being proximal to the cofactor FAD and to the putative substrate binding site (Fig. 6a). Finally, the original CASTing library, MAO-N D5 and MAO-N D5 T93F/466T, were analysed under the assay conditions after incubation with MBEA. Due to low levels of substrate uptake by the cells, 1% (v/v) butan-1-ol was added at the time of substrate addition to increase the permeability of the *E. coli* cell membranes to the substrate during the assay, resulting in sufficient uptake of MBEA to observe a marked increase in fluorescence of cells expressing MAO-N D5 T93F/466T relative to the original CASTing library and MAO-N D5 (Fig. 6b).

## Discussion and conclusion

As a reactive oxygen species (ROS), hydrogen peroxide (H_2_O_2_) is generated naturally through numerous cellular processes. In eukaryotes, production of H_2_O_2_ can be indicative of oxidative stress and, as such, multiple fluorescent probes have been developed to act as oxidative stress indicators.^47^,^59^ In this study, we utilise one such probe (C-H_2_DCFDA) to assay directly the activity of a recombinantly expressed amine oxidase within an *E. coli* cell. Under the optimised assay conditions, it is clear that cells expressing MAO-N D5 have a substrate and time-dependent fluorescence. As with most bacterial cultures, there is considerable heterogeneity, and more than can be ascribed merely to variation in the cell cycle.^60^–^65^ Nonetheless, through iterative rounds of FACS, it was possible to enrich libraries with amine oxidase variants with improved function. We have demonstrated the utility of this assay by identifying a novel, double mutant of MAO-N D5, which shows oxidase activity toward a secondary amine substrate which was not oxidised by the parent enzyme. By testing relevant ROS probes we could rapidly identify those capable of detecting intracellular MAO-N activity. However, considerable optimisation of the assay conditions and cell treatment was required to detect, sort and recover enzyme variants with improved catalytic activity reliably. Conditions such as *E. coli* strain, buffer constituents, reaction quenching and PCR recovery of sorted populations proved pivotal in obtaining a robust protocol capable of rapid detection of oxidase activity. By identifying a new MAO-N variant with activity towards a novel substrate we demonstrate its use in a directed evolution study, providing a quick and sensitive means to screen large variant libraries and enrich variants exhibiting a desired catalytic activity. While oxidase activity has been detected using yeast in emulsion droplets,^43^,^44^,^66^ to our knowledge this is the first demonstration that oxidase activity from recombinant protein can be assayed within a single prokaryotic cell and used to enrich enzyme variants exhibiting improved activity.

## Conflicts of interest

There are no conflicts of interest to declare.

## Supplementary Material

Supplementary information
