# Supplementary material for: Ultra-high throughput functional enrichment of large monoamine oxidase (MAO-N) libraries by fluorescence activated cell sorting
Source: Analyst. 2018 Sep 10;143(19):4747–55. doi: 10.1039/c8an00851e (PMC6156879; doi:10.1039/c8an00851e)
Supplement: Supplementary file 1 [file AN-143-C8AN00851E-s001.pdf]

Electronic Supplementary Material (ESI) for Analyst.
This journal is © The Royal Society of Chemistry 2018

Ultra-high throughput functional enrichment of large monoamine oxidase (MAO-N) libraries by fluorescence activated cell sorting

Supporting Information

[1,2,3]Joanna C. Sadler, Andrew Currin & [1,2,3,*]Douglas B. Kell

[1]School of Chemistry, [2]The Manchester Institute of Biotechnology, [3]Centre for the Synthetic Biology of Fine and Speciality Chemicals (SYNBIOCHEM), The University of Manchester, 131 Princess St, Manchester M1 7DN, UK.

joanna.sadler@manchester.ac.uk, dbk@manchester.ac.uk

*corresponding author http://dbkgroup.org/ @dbkell

Contents

Effect of freeze-thaw step on probe uptake..2

Comparison between K12 and B strains of *E. coli* 3

Identification of an assay quenching agent..4

Effect of freeze-thaw step on probe uptake

(a)

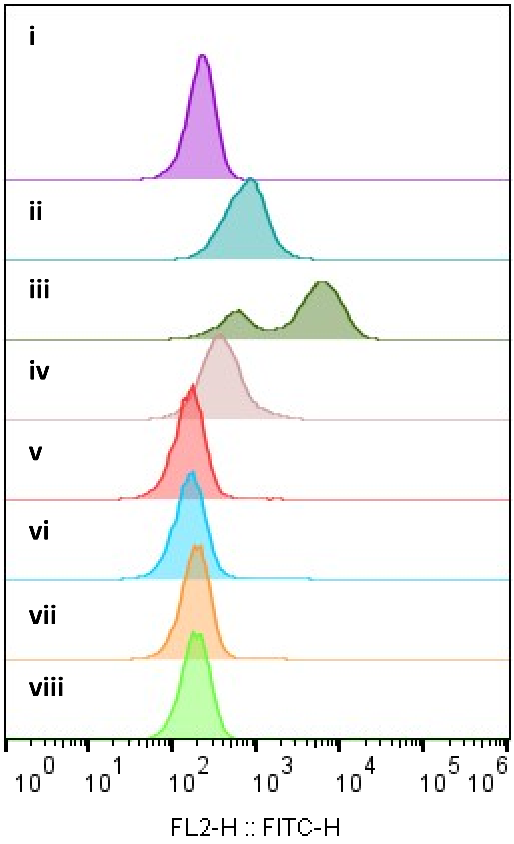

(b)

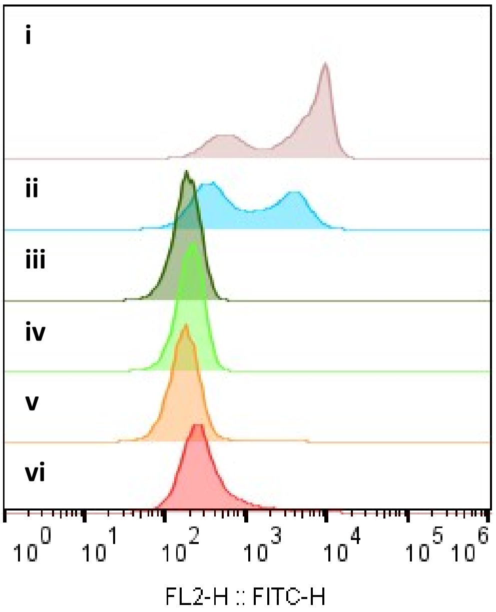

(a)	Sample	Population	Mean FITC-H	FITC-H CV
i	Induced freeze-°thawed cells only	Total gated	232	36
ii	Induced freeze-thawed + probe	Total gated	861	56
iii	Induced freeze-thawed + probe + AMBA	Positive	6512	51
iv	Induced Non-induced freeze-thawed + probe + AMBA	Total gated	464	74
v	Induced + probe	Total gated	183	98
vi	Induced + probe + substrate	Total gated	202	155
vii	Non-Induced + probe	Total gated	217	81
viii	Non-Induced + probe + substrate	Total gated	222	94

(b)	Sample	Population	Mean FITC-H	FITC-H CV
i	Induced freeze-thawed + probe + H_2O_2	Positive	7240	42
ii	Non-Induced freeze-thawed + probe + H_2O_2	Total gated	3591	53
iii	Induced freeze-thawed + H_2O_2	Positive	189	38
iv	Non-Induced freeze-thawed + H_2O_2	Total gated	208	37
v	Induced + probe + H_2O_2	Total gated	222	181
vi	Non-induced + probe + H_2O_2	Total gated	492	223

Figure S1. (a) Comparison between the fluorescence of induced and non-induced *E. coli* cells expressing MAO-N before and after a freeze-thaw step. (b) Effect of freeze-thaw step on probe uptake of induced and non-induced cells determined by spiking with 10 µM H_2O_2.

Comparison between K12 and B strains of *E. coli*

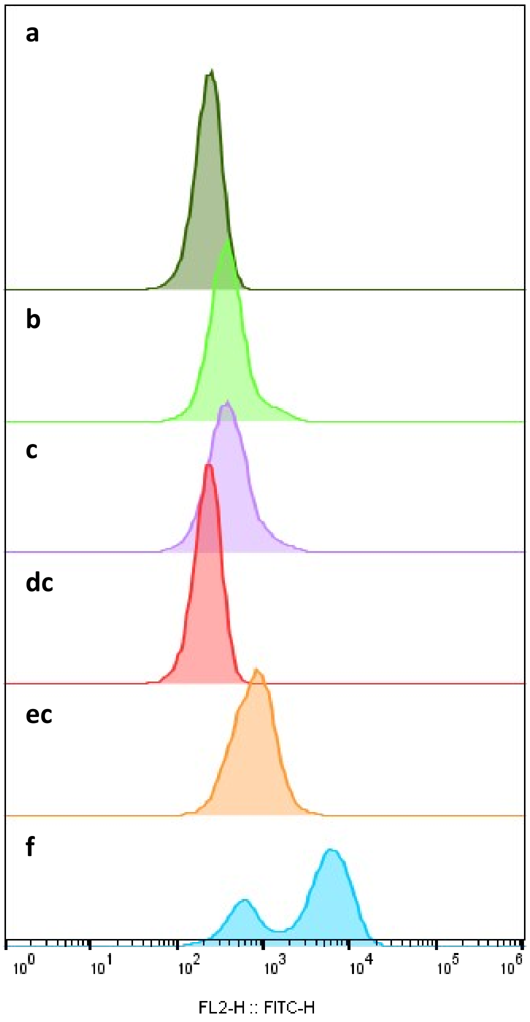

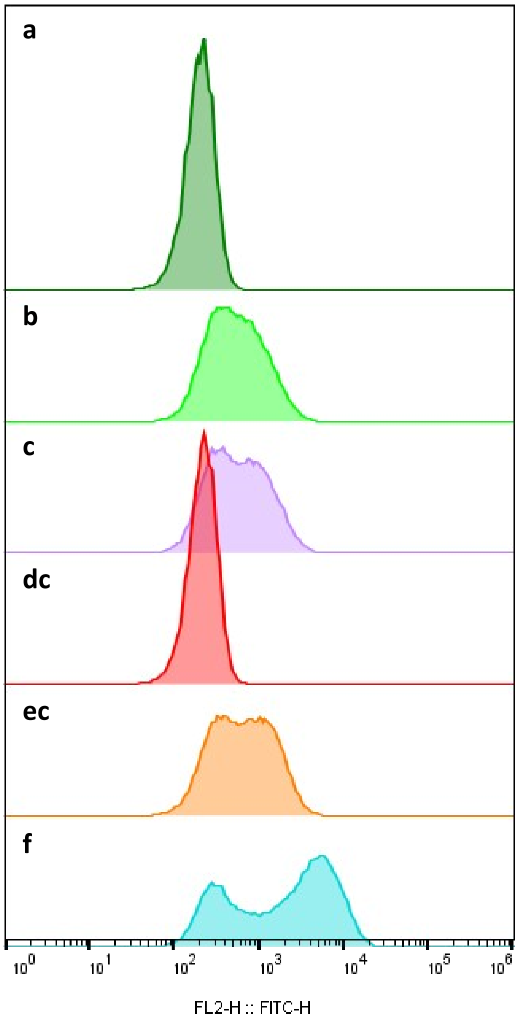

	Sample	Population	Mean FITC-H	FITC-H CV
a	T7 Non-Induced	Total gated	237	36
b	T7 Non-Induced + probe	Total gated	445	69
c	T7 Non-Induced + probe + AMBA	Positive	464	74
d	T7 Induced	Total gated	232	36
e	T7 Induced + probe	Total gated	861	56
f	T7 Induced cells + probe + AMBA	Positive	6512	51

	Sample	Population	Mean FITC-H	FITC-H CV
a	5-alpha Non-Induced	Total gated	211	40
b	5- alpha Non-Induced + probe	Total gated	694	84
c	5- alpha Non-Induced + probe + AMBA	Positive	758	86
d	5- alpha Induced	Total gated	227	38
e	5- alpha Induced + probe	Total gated	859	84
f	5- alpha Induced cells + probe + AMBA	Positive	5340	68

Figure S2. Comparison between (a) T7 express (B strain) and (b) 5-alpha cells (K12 strain) as hosts for the oxidase activity assay. For detection of B strain cells, it was necessary to reduce the FSC threshold to 1.5%.

Identification of an assay quenching agent

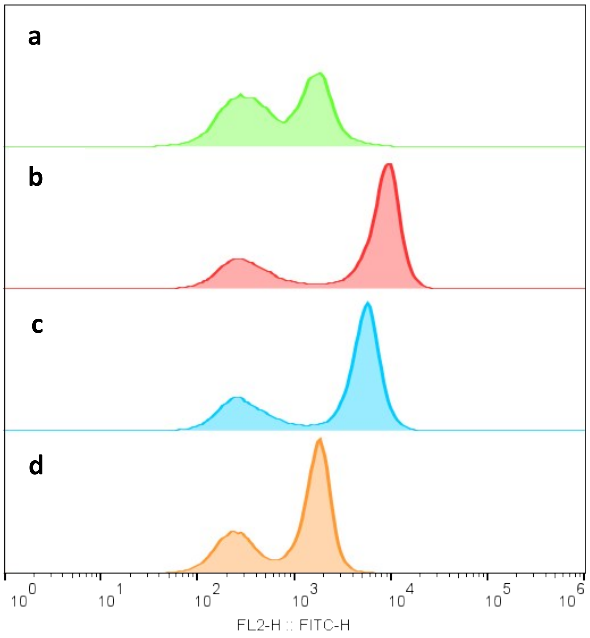

	Sample	Population	Mean FITC-H	FITC-H CV
**a**	No quench, t=5	Positive	1721	40
**b**	No quench; t=30	Positive	8716	38
**c**	10 mM sodium pyruvate at t=5[a]	Positive	5589	38
**d**	10 mM L-ascorbic acid at t=5[a]	Positive	1739	36

Figure S3. Fluorescence of E. coli cells under the optimised assay conditions after (a) 5 minutes incubation with AMBA; (b) 30 minutes incubation with AMBA; (c) quenching with 10 mM sodium pyruvate after 5 minutes incubation with AMBA; (d) quenching with 10 mM ascorbic acid after 5 minutes incubation with AMBA. [a]Cells were analysed 30 minutes after quenching.

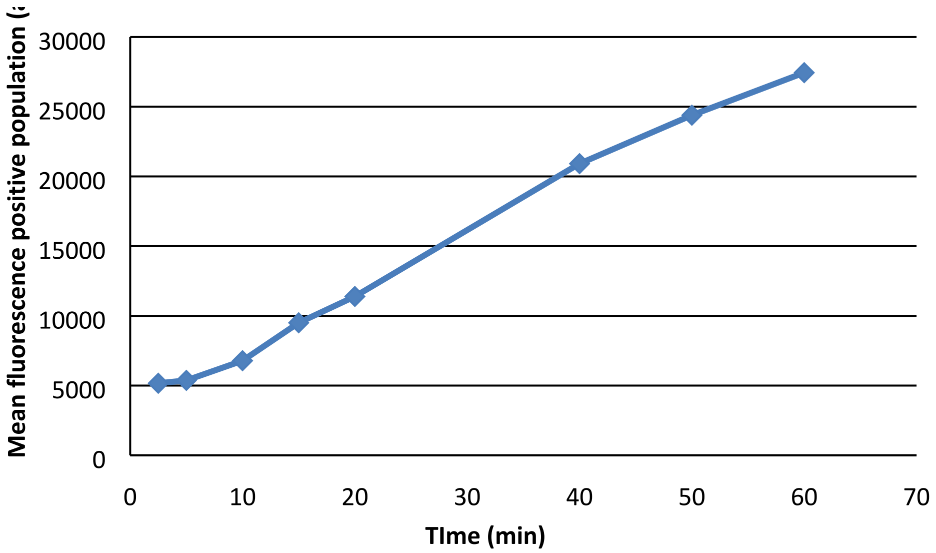

Figure S4. Mean fluorescence of E. coli cells under the optimised assay conditions quenched with 10 mM ascorbic acid at different timepoints after addition of AMBA.
